# Serum PBDEs in a North Carolina Toddler Cohort: Associations with Handwipes, House Dust, and Socioeconomic Variables

**DOI:** 10.1289/ehp.1104802

**Published:** 2012-05-23

**Authors:** Heather M. Stapleton, Sarah Eagle, Andreas Sjödin, Thomas F. Webster

**Affiliations:** 1 Nicholas School of the Environment, Duke University, Durham, North Carolina, USA; 2 Centers for Disease Control and Prevention, National Center for Environmental Health, Division for Laboratory Sciences, Atlanta, Georgia, USA; 3 Department of Environmental Health, Boston University School of Public Health, Boston, Massachusetts, USA

**Keywords:** children, exposure, handwipes, house dust, PBDEs

## Abstract

Background: Polybrominated diphenyl ethers (PBDEs) are persistent, bioaccumulative, and endocrine-disrupting chemicals.

Objectives: We used handwipes to estimate exposure to PBDEs in house dust among toddlers and examined sex, age, breast-feeding, race, and parents’ education as predictors of serum PBDEs.

Methods: Eighty-three children from 12 to 36 months of age were enrolled in North Carolina between May 2009 and November 2010. Blood, handwipe, and house dust samples were collected and analyzed for PBDEs. A questionnaire was administered to collect demographic data.

Results: PBDEs were detected in all serum samples (geometric mean for ΣpentaBDE in serum was 43.3 ng/g lipid), 98% of the handwipe samples, and 100% of the dust samples. Serum ΣpentaBDEs were significantly correlated with both handwipe and house dust ΣpentaBDE levels, but were more strongly associated with handwipe levels (*r* = 0.57; *p* < 0.001 vs. *r* = 0.35; *p* < 0.01). Multivariate model estimates revealed that handwipe levels, child’s sex, child’s age, and father’s education accounted for 39% of the variation in serum ΣBDE_3_ levels (sum of BDEs 47, 99, and 100). In contrast, age, handwipe levels, and breast-feeding duration explained 39% of the variation in serum BDE 153.

Conclusions: Our study suggests that hand-to-mouth activity may be a significant source of exposure to PBDEs. Furthermore, age, socioeconomic status, and breast-feeding were significant predictors of exposure, but associations varied by congener. Specifically, serum ΣBDE_3_ was inversely associated with socioeconomic status, whereas serum BDE-153 was positively associated with duration of breast-feeding and mother’s education.

Polybrominated diphenyl ethers (PBDEs) are flame retardant chemicals that have been used to reduce the flammability of polymers and resins found in commercial products such as furniture, electronics (e.g., TVs, cell phones, business equipment), and electrical wiring and in some textile applications. Historically three different commercial mixtures, known as pentaBDE, octaBDE, and decaBDE, were sold by different companies under different trade names. However, the persistence, bioaccumulation, and potential toxicity of components in the pentaBDE and octaBDE mixtures resulted in their phase out in many regions of the world, including the United States (Tullo 2003).

PBDE congeners found in the pentaBDE commercial mixture are commonly found in human tissues (Schecter et al. 2005; Sjödin et al. 2003, 2008). This mixture was used primarily in polyurethane foam found in furniture, and most of the pentaBDE used in furniture was manufactured in the United States and Canada (Hale et al. 2003). The high use of pentaBDE in North America may explain why these populations have some of the highest PBDE levels ever recorded in non-occupationally exposed populations (Hites 2004). In the 2003–2004 NHANES (National Health and Nutrition Examination Survey) survey of the U.S. population (≥ 12 years of age), > 97% of the population had detectable levels of PBDE in their blood. Concentrations in 12- to 19-year-olds were higher than in older age groups, suggesting that adolescents experienced a higher exposure level than adults (Sjödin et al. 2008). More recent studies have examined PBDE levels in younger children (Rose et al. 2010; Roze et al. 2009; Toms et al. 2008; Windham et al. 2010). Rose et al. (2010) measured PBDEs in serum from 2- to 5-year-old children in California and found that their levels were 2–10 times higher than most adults in the US.

Children are likely exposed to PBDEs from many sources. Exposure modeling suggests that most exposures occur from diet, inhalation, and contact with contaminated house dust (Jones-Otazo et al. 2005; Lorber 2008). House dust is often contaminated with numerous chemicals, and previous studies have linked children’s exposure to lead with exposure from contaminated house dust (Butte and Heinzow 2002; Rhoads et al. 1999). PBDEs are often detected in house dust and they have also been measured in handwipe samples (Rudel et al. 2003; Stapleton et al. 2008). This suggests that children with more hand-to-mouth contacts may also be more exposed to contaminants from their hand-to-mouth activities. In U.S. adults, both diet and exposure to dust have been shown to be associated with pentaBDE body burdens (Fraser et al. 2009; Wu et al. 2007); however, more recently, studies in the United States suggest that dust exposure may have more of an influence than diet on serum PBDE levels (Johnson et al. 2010; Watkins et al. 2012). The most significant sources of PBDE exposure in children are currently unknown; however, dust and diet are both hypothesized to play major roles, although such estimates are complicated by the uncertainty surrounding exposure factors for dust ingestion (Lorber 2008; Rose et al. 2010).

Children’s exposure to PBDEs is of concern because of PBDEs’ known endocrine-disrupting and neurodevelopmental properties as observed in animal studies. PBDEs have chemical structures similar to thyroid hormones, such as thyroxine. This similarity in structure may explain why PBDE exposure in animals has been linked to significant changes in circulating thyroid hormone levels (Fernie et al. 2005; Tomy et al. 2004; Zhou et al. 2002). In addition, *in vitro* studies have shown that PBDEs and their oxidative metabolites are potent competitors with thyroid hormones for binding to transthyretin (Meerts et al. 2000) and human type 1 deiodinase, which metabolizes thyroid hormones (Butt et al. 2011). PBDEs and their metabolites have also been shown to affect spontaneous behavior in rodents and affect cholinergic function and calcium signaling in the brain (Dingemans et al. 2010a, 2010b; Viberg et al. 2002, 2003). Recent human studies have also reported significant associations between serum PBDEs and altered thyroid hormone levels in adults (Turyk et al. 2008) and pregnant women (Chevrier et al. 2010; Stapleton et al. 2011), and with motor, cognitive, and behavioral performances in children (Herbstman et al. 2010; Roze et al. 2009).

This study was undertaken to better understand the variables contributing to PBDE exposure in young children, specifically toddlers between 1 to 3 years of age, who have the highest hand-to-mouth activities (Tulve et al. 2002). Our primary objective was to estimate the influence of indoor exposure from house dust on serum PBDE levels in toddlers. A secondary objective was to explore the relationships between PBDE exposures and socioeconomic status (SES; e.g., race/ethnicity and parental education), because some researchers have suggested that SES may contribute to exposure disparities (Zota et al. 2010).

## Materials and Methods

*Participant recruitment.* All aspects of this study were authorized by the Duke University Institutional Review Board before initiation, and all parents/guardians gave informed consent before sample collection. Recruitment was conducted through a pediatric clinic in Durham, North Carolina, and by letters mailed to patients of three local pediatric clinics in Durham. Families with children between the ages of 12–36 months, with no prior diagnoses of thyroid problems, were recruited for this study. Recruitment began in May 2009 and continued through November 2010.

*Sample collection.* From every child a small blood sample (~ 4 mL blood) was collected by venipuncture. Children recruited through the pediatric clinic (*n* = 9) had a blood sample collected during their doctor’s appointment by laboratory staff. Participants recruited after responding to letters (*n* = 74) had their blood collected by a contract phlebotomist during the home visit. Every family participating in the study received a visit from our research team. During the home visit (between 0900 and 1700 hours) each family filled out a short questionnaire administered by a research staff member who collected information on the child and information on the parents’ education levels. A handwipe sample was collected from each child and a house dust sample was collected from the area of the home identified by the parent/guardian as the area in which the child spent the most active time. Handwipe and dust samples were collected using published methods (Allen et al. 2008a; Stapleton et al. 2008). Briefly, for handwipe sample collections, sterile gauze wipes were first soaked in exactly 3.0 mL isopropyl alcohol. The entire surface area of the child’s hands (including top and bottom of fingers) was then wiped two times from fingers to the wrist. All wipes were investigator collected and then stored in pre-cleaned scintillation vials at –4°C until analysis. A Eureka Mighty-Mite vacuum cleaner (Model 3670) and crevice tool attachment were used to collect dust samples. Dust was captured by a cellulose extraction thimble (Whatman International, Maidstone, UK) inserted between the crevice tool and the vacuum tube extender and secured using a rubber O-ring. The equivalent of the entire floor-surface area for the room was vacuumed by gently drawing the crevice tool across the top of all surfaces. Cellulose thimbles used to collect the dust were removed from the vacuum, wrapped in foil, and stored in plastic bags at –4°C until analysis.

*Chemicals.* Individual PBDE congeners used for analysis of the handwipes and house dust samples were purchased from AccuStandard, Inc. (New Haven, CT). ^13^C-2,2´,3,4,5,5´-hexachlorodiphenylether (CDE-141) and ^13^C-decabromodiphenylether (^13^C-BDE-209) were purchased from Wellington Labs (Guelph, ON, Canada) and used as a recovery and internal standard, respectively. 4-Fluoro-2,3,4,6-tetrabromodiphenylether (FBDE-69), purchased from Chiron (Trondheim, Norway), was used as an internal standard. All solvents and other materials were HPLC grade or better.

*Serum analysis.* Blood samples were transferred to the laboratory on ice packs in a cooler and allowed to sit for 1–2 hr to facilitate clotting. They were then centrifuged at 3,000 rpm for 5 min to isolate the serum. Serum was transferred to pre-cleaned amber bottles and frozen at –20°C until analysis. Serum samples were analyzed for tri- to decaBDEs (11 congeners) and lipids at the Centers for Disease Control and Prevention (CDC) using established methods (Sjödin et al. 2004, 2008).

*Handwipe analysis.* The wipes were extracted and analyzed for PBDE congeners using a method reported by Stapleton et al. (2008). Handwipe samples were spiked with both FBDE-69 and ^13^C-BDE-209 before extraction as both a surrogate and internal standard.

*Dust analysis.* Dust samples were sieved to < 500 µm and then extracted and analyzed for PBDE congeners using a method reported by Allen et al. (2008a). Dust samples were spiked with both FBDE-69 and ^13^C-BDE-209 before extraction as both a surrogate and internal standard.

*Quality control.* Clean gauze pads and sodium sulfate were used as laboratory blanks for the handwipes and house dust, respectively. SRM 2585 (Organic Contaminants in House Dust; National Institute of Standards and Technology, Gaithersburg, MD) was used for quality assurance, to test performance of the dust extraction method for PBDEs. Handwipe and house dust PBDE measurements were blank subtracted using the average field (handwipe) or laboratory (house dust) blank measurement. BDEs 47, 99, 100, and 209 were routinely detected in the blanks, although at levels that were typically much lower (e.g., < 1% in dust and < 10% in wipes) than those of the samples, with values ranging from less than detection to a maximum of 4.6 ng for BDE-209. The method detection limits (MDL) for each sample type were determined as three times the standard deviation of the appropriate blanks after subtracting the average blank level, and normalized to average extraction mass of the sample. The laboratory instrument detection limit was used as the MDL (i.e., signal-to-noise response ratio of 3) in instances where congeners were not detected. Recovery of the internal standard, FBDE-69, averaged 102 ± 15% in the handwipe samples, and 86 ± 25% in the house dust samples. Recovery of ^13^C-BDE-209 in the handwipe extracts was variable for unknown reasons. It was detected in only 28% of the samples and averaged 56 ± 31% when detected. Recovery of the tri–decaBDE congeners in the dust SRM 2585 ranged from 85% to 133%.

*Statistical analysis.* Values < MDL were assigned a value equal to half the detection limit for statistical analyses. PBDE measurements are reported in mass terms instead of molar units, because mass is more commonly used in the literature. Summary statistics were only computed for congeners with detection frequencies of ≥ 50%. To allow for comparison of serum, handwipes, and dust using the same congeners, we refer to ΣpentaBDE throughout this paper as the sum of BDEs 47, 99, 100 and 153, and ΣBDE_3_ represents the sum of BDEs 47, 99, and 100. ΣBDE_3_ was calculated separately due to the different behavior of BDE-153 relative to BDEs 47, 99, and 100 observed in correlation and multivariate analyses. The distributions of PBDEs were assessed using histograms, Q-Q plots and Shapiro–Wilk tests and measured PBDE values were log-transformed for analysis as distributions were much closer to log-normal than normal. Regression analyses were checked to confirm that residuals were homoscedastic and normally distributed. Pearson correlation coefficients were calculated using log-transformed data. Univariate and multivariate models were used to examine predictors of log-transformed serum ΣBDE_3_ and BDE-153. Variables examined included children’s sex, age (continuous variable), race, parents’ education levels (a measure of SES), duration of breast-feeding (continuous variable), time children spent away from home (hours/week), dust concentrations, and handwipe levels. Education was dichotomized as having a completed college degree (associates, bachelor’s, or graduate) or not. Parents/guardians declared their child’s race after reviewing five different choices: American Indian/Alaskan Native, Asian, Native Hawaiian or Pacific Islander, Black/African American, or White. Due to low sample numbers in nonwhite categories, race was then categorized as white or nonwhite. Parents/guardians initially categorized their children as either Hispanic or non-Hispanic in the questionnaire. Preliminary analysis showed no significant differences by ethnicity (data not shown), so this distinction was dropped. When a father’s education was listed as unknown (*n* = 9), it was imputed as no college degree. Handwipe and dust ΣBDE_3_ and BDE-153 levels were categorized as tertiles. This allows for the analysis of trends without imposing linearity and reduces the influence of outlier points. Categories for handwipes for ΣBDE_3_ were low/reference (< 19.0 ng/wipe), medium (19.0 < *x* < 60.75 ng/wipe), high (> 60.75 ng/wipe). Categories for dust were low/reference (< 1027.5 ng/g), medium (1027.5 < *x* < 3,040 ng/g), high (> 3,040 ng/g). Categories for handwipes for BDE-153 were low/reference (< 0.6 ng/wipe), medium (0.6 < *x* < 1.6 ng/wipe), high (> 1.6 ng/wipe). Categories for dust were low/reference (< 40 ng/g), medium (40 < *x* < 130 ng/g), high (> 130 ng/g). Exponentiated regression coefficients from models of log-transformed serum PBDE concentrations represent the percent change in the geometric mean (GM) concentration associated with exposure. Statistical significance was set at α < 0.05.

## Results

*Population characteristics.* Table 1 presents the characteristics of the toddlers enrolled in this study. Participants were reasonably distributed across sex, age, race, duration of breast-feeding, and parents’ education. After enrollment and blood collection, one individual dropped out from the study and was unavailable for the home visit; hence, handwipe and dust samples were unavailable for this subject. Seven of the 83 fully enrolled individuals did not have blood collected because of collection complications. This resulted in a total of 77 serum samples, 83 handwipe samples, and 81 dust samples collected during this study.

**Table 1:** Sample population characteristics (n = 83).

Characteristic	n (%)	
Child’s sex		
Female	42	(51)
Male	41	(49)
Child’s age (months)		
11–18	26	(31)
19–24	34	(41)
25–36	23	(28)
Child’s race/ethnicity		
Hispanic	16	(19)
Non-Hispanic black	21	(25)
Non-Hispanic white	43	(52)
Other	3	(4)
Marital status of parents		
Married	55	(66)
Unmarried	28	(34)
Mother’s education level		
Some high school	8	(10)
Completed high school	8	(10)
Some college	12	(14)
Completed college	25	(30)
Graduate degree	30	(36)
Father’s education level		
Some high school	10	(12)
Completed high school	11	(13)
Some college	7	(8)
Completed college	18	(22)
Graduate degree	28	(34)
Not reported	9	(11)
Breast-feeding		
< 1 month	20	(27)
1–6 months	21	(28)
> 6 months	34	(45)
Hours/week away from home		
0–12	33	(40)
12–36	24	(29)
> 36	26	(31)


*PBDEs in serum.* PBDEs were detected in all 77 serum samples. The most frequently detected congeners (i.e., detected in > 50% of the samples) were BDEs 47, 99, 100, and 153. BDEs 17, 28, 66, 85, 154, 183, and 209 were also detected in some samples but with lower frequencies (Table 2). BDE-47 was the largest contributor to the ΣpentaBDE concentration measured, ranging from 18% to 72% of the total in individual samples, and averaging 52% overall. BDEs 153, 99, and 100 contributed on average 16%, 15%, and 11% of the ΣpentaBDE concentration. Among the 77 serum samples analyzed, ΣpentaBDE ranged from 5.2 to 668 ng/g lipid with a GM of 42.9 ng/g lipid.

**Table 2:** Levels of PBDE congeners measured in serum, handwipes, and house dust.

Serum (ng/g lipid), n = 77				Handwipes (ng), n = 83				House dust (ng/g), n = 81			
Congener	% Detect	GM	Range	% Detect	GM	Range	% Detect	GM	Range
BDE-17		7.8	NA		< 0.4–5.3		3.5	NA		< 0.5–3.3		58	2.7		< 1.2–94
BDE-28		26	NA		< 0.4–11.7		45	NA		< 0.5–14.8		90	11		< 1.5–277
BDE-47		97	23.3		< 3.0–350		94	16.3		< 2.3–923		100	870		55–24,720
BDE-66		3.9	NA		< 0.4–4.5		52	0.32		< 0.2–67.8		90	14		< 1.1–916
BDE-85/155		47	NA		< 0.4–10.3		72	0.55		< 0.2–27.7		96	38		< 0.7–1,860
BDE-99		99	6.39		< 1.1–225		88	13.8		< 3.1–1,001		100	919		8–36,210
BDE-100		95	4.97		< 0.5–67.2		82	3.2		< 0.9–228		100	176		9–10,230
BDE-153		96	5.34		< 0.5–83.1		82	1.0		< 0.3–35.3		100	88		7–3,407
BDE-154		38	NA		< 0.4–17.1		87	0.96		< 0.3–46.6		100	74		5–3,061
BDE-183		18	NA		< 0.4–1.7		1.1	NA		< 1.8–2.1		85	8.6		< 1.8–162
BDE-209		17	NA		< 6.0–63.8		27a	NA		< 4.5–283		100	2,574		441–76,130
∑BDE3b		99	35.2		4.5–642		95	33.7		3.2–2,152		100	2,057		145–71,150
∑PentaBDEc		100	42.9		5.2–668		98	35.0		3.3–2,187		100	2,153		152–74,560
Abbreviations: % Detect, percent detectable; NA, not available. aDetection compromised by low recovery of 13C-BDE-209 during extraction. b∑BDE3 represents the sum total of BDE congeners 47, 99, and 100. c∑PentaBDE represents the sum total of BDE congeners 47, 99, 100, and 153.															

*PBDEs in handwipes.* PBDEs were detected in 81 of the 83 handwipe samples analyzed. The most frequently detected congeners (i.e., detected in > 50% of the samples) were BDEs 47, 66, 85/155, 99, 100, 153, and 154. BDEs 17, 28, 183, and 209 were also detected at lower frequencies (Table 2). BDEs 47 and 99 were the largest contributors to the total PBDE burden in the handwipes, averaging 38% and 33%, respectively, in individual samples. BDE-209 was detected in 27% of the handwipe samples; however, recovery of ^13^C-BDE-209 was very low in some samples (< 50% in 72% of the samples), and this may have caused a reduced detection frequency that may have biased our results.

*PBDEs in house dust.* PBDEs were detected in all house dust samples analyzed. All major PBDE analytes were detected in > 50% of the samples (Table 2). BDEs 209, 99, and 47 were the largest contributors to the total PBDE concentration in the house dust averaging 48%, 19%, and 18%, respectively. BDE-209 had the highest average concentration in the dust samples, ranging from 441 to 76,130 ng/g with a GM of 2,574 ng/g.

**Figure 1:**
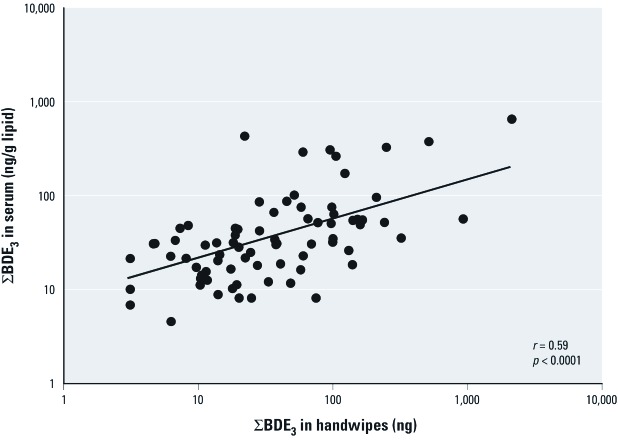
Correlation between serum ∑BDE_3_ and handwipe ∑BDE_3_.

*Correlations between PBDEs in serum, handwipes and dust.* Pearson correlation coefficients were calculated for individual log transformed BDE congeners within and between serum, handwipes, and house dust samples [see Supplemental Material, Table S1 (http://dx.doi.org/10.1289/ehp.1104802)]. PBDEs were highly correlated within each matrix, with correlation coefficients of 0.61 (*p* < 0.0001) or higher. BDEs 47, 99, and 100 were highly correlated (*r* > 0.9) with each other within serum, whereas BDE-153 displayed lower correlation coefficients (*r* = 0.5– 0.7) with the other congeners in serum. The highest correlations between matrices were between serum and handwipes for ΣBDE_3_ (*r* = 0.59; *p* < 0.0001; Figure 1). In contrast the association between BDE-153 in serum and handwipes was lower, although still significant: *r* = 0.31 (*p* = 0.007). Correlations between handwipes and dust were similar for both ΣBDE_3_ and BDE-153: 0.36 (*p* = 0.001) and 0.34 (*p* = 0.003) respectively. In addition, the correlation of ΣBDE_3_ between serum and dust, *r* = 0.34 (*p* = 0.003) was less than that for serum and handwipes, but greater than the association of BDE-153 in serum and dust: *r* = 0.19 (*p* = 0.09). We therefore report results separately for ΣBDE_3_ and BDE-153 given that patterns of exposure appear to differ.

*Predictors of ΣBDE_3_ in children’s serum.* Table 3 shows the results of regression of log-transformed serum ΣBDE_3_ with characteristics of the child and their parents, as well as by ΣBDE_3_ levels measured in handwipes and dust (by tertiles). Univariate estimates indicate that serum levels tended to increase with age (34%/year, *p* = 0.31) and were 45% higher in males than females (*p* = 0.12), although neither result was significant. Neither months of breast-feeding nor time away from home (hours/week) were significant predictors. Serum concentrations were strongly and inversely associated with father’s education, with levels about half as high in children whose fathers had college educations as those without (*p* = 0.003). Mother’s education and race (both related to father’s education) also predicted children’s serum levels. Because father’s education level was a slightly stronger predictor, we used this variable in the multivariate analysis. Serum ΣBDE_3_ levels increased with higher tertiles of ΣBDE_3_ levels in handwipes, with serum concentrations in the high handwipe group 3.66 times higher than those in the low group (*p* < 0.0001). Dust ΣBDE_3_ concentrations were not as strong a predictor of serum levels as handwipe levels, but still showed an increasing trend (serum concentrations 1.78 times higher in the high dust group compared to the low dust group; *p* = 0.05).

**Table 3:** Regression analysis of predictors of PBDE concentrations in serum.

∑BDE^3^							BDE-153						
Univariate			Adjusted			Univariate			Adjusted
Predictor	Coefficient^a^ (95% CI)	p-Value	Coefficient^a^ (95% CI)	p-Value	Coefficient^a^ (95% CI)	p-Value	Coefficient^a^ (95% CI)	p-Value
Age (years)		1.34 (0.76, 2.38)		0.31		1.61 (0.99, 2.62)		0.05		2.41 (1.38, 4.22)		0.002		1.73 (1.05, 2.84)		0.03
Child sex																
Female (referent)		1				1				1						
Male		1.45 (0.90, 2.31)		0.12		1.34 (0.90, 2.00)		0.15		1.37 (0.84, 2.23)		0.21				
Father’s education																
No college degree (referent)		1				1				1						
College degree		0.50 (0.31, 0.78)		0.003		0.48 (0.32, 0.74)		0.001		1.53 (0.94, 2.51)		0.09				
Mother’s education																
No college degree (referent)		1								1						
College degree		0.62 (0.38, 1.03)		0.07						1.80 (1.07, 3.03)		0.03				
Child’s race																
Nonwhite (referent)		1								1						
White		0.53 (0.33, 0.84)		0.008						1.19 (0.72, 1.97)		0.48				
Handwipe																
Low (referent)		1				1				1				1		
Medium		1.83 (1.11, 3.02)		0.02		1.62 (1.01, 2.61)		0.048		1.70 (0.95, 3.04)		0.07		1.85 (1.13, 3.03)		0.01
High		3.66 (2.22, 6.05)		< 0.0001		2.94 (1.80, 4.79)		< 0.0001		2.41 (1.37, 4.24)		0.003		2.44 (1.51, 3.95)		0.0004
Dust																
Low (referent)		1								1						
Medium		1.24 (0.70, 2.19)		0.45						1.34 (0.73, 2.48)		0.34				
High		1.78 (1.01, 3.15)		0.05						1.35 (0.75, 2.46)		0.31				
Breast-feeding (months)		0.99 (0.95, 1.02)		0.41						1.07 (1.04, 1.11)		< 0.0001		1.07 (1.04, 1.10)		< 0.0001
Away from home (hr/week)		1.00 (0.98, 1.01)		0.50						1.00 (0.99, 1.01)		0.95				
aExponentiated beta-coefficients represent the multiplicative change in the serum concentration relative to the reference group for categorical variables, or per unit change for continuous variables (age, breast-feeding duration, time away from home).																

A multivariate model including age, sex, father’s education, and handwipes indicated a stronger association between serum ΣBDE_3_ and age (61%/year, *p* = 0.05), little change in associations with sex or father’s education, and slightly weaker associations with ΣBDE_3_ levels in handwipes, although the latter remain strongly associated with serum levels. The explained variation in this multivariate model (*r*^2^) was 0.39. Models that also included dust ΣBDE_3_ concentrations showed a weaker association with dust concentrations compared with the univariate model estimates, but little change in associations with handwipe concentrations from univariate estimates (data not shown), suggesting that handwipe concentration is in part an intermediate variable.

*Predictors of BDE-153 in children’s serum.* In a univariate analysis of BDE-153 in children’s serum (Table 3), age was strongly associated with increased concentrations (141%/year, *p* = 0.002). In contrast with ΣBDE_3_, duration of breast-feeding was also a very strong predictor (7%/month, *p* < 0.0001) of serum BDE-153 levels. In this cohort, approximately 34% of the children were breast-fed for > 12 months, up to a maximum of 27 months. Also in contrast with ΣBDE_3_, BDE-153 in serum was positively (rather than negatively) associated with socioeconomic status variables, with mother’s education being the strongest predictor (80% increase in children whose mothers had a college degree, *p* = 0.03). Handwipe BDE-153 levels were also associated with serum BDE-153, although associations were not as strong as those between handwipe and serum ΣBDE_3_ levels. Consistent with the earlier correlation results, dust (categorized) was not a strong univariate predictor.

Duration of breast-feeding and mother’s education were highly associated (data not shown), with the former being the most significant predictor of BDE-153, so it was retained for multivariate analysis. The multivariate results show strong associations between serum BDE-153 concentrations and age (73%/year, BDE-153, 0.03), duration of breast-feeding (7%/month, *p* < 0.0001), and handwipe BDE-153 levels. Addition to this model of maternal education largely eliminated the association with breast-feeding, but had little effect on the coefficients for the other variables (data not shown); these results suggest that breast-feeding may mediate the effect of maternal education on children’s serum BDE-153, but other explanations cannot be ruled out. The effect of handwipe levels showed an increasing trend across categories, with serum concentrations 2.44 times higher in the high versus low handwipe categories (*p* = 0.0004). The explained variation in this model (*r*^2^) was 0.39.

## Discussion

PBDE serum levels measured here in 1- to 3-year-olds are comparable to levels reported among the general U.S. population ≥ 12 years of age in the 2003–2004 NHANES (Sjödin et al. 2008). However, pentaBDE was voluntarily phased out from use in the United States at the end of 2004, so we would expect to observe declining PBDE levels in the U.S. population in years following. Watkins et al. (2011) measured the same PBDE congeners (using the same laboratory) in serum collected from a cohort of 31 adult Boston, Massachusetts, office workers in 2009. The geometric mean level of BDE-47 in their cohort was 14.2 ng/g lipid, significantly (*p* < 0.05) lower than measured in our toddler cohort (23.3 ng/g lipid). Although our cohort’s levels are higher than those of adults, they are lower than levels reported in a California cohort of 2- to 5-year-olds sampled between 2003 and 2005, 4–7 years before our cohort (Rose et al. 2010).

The current results in toddlers—associations between ΣBDE_3_ concentrations in handwipes, dust, and serum—support an important role for exposure via the indoor environment, which is likely occurring through hand-to-mouth activity. The correlation between ΣBDE_3_ in serum and handwipes (*r* = 0.59) was stronger than the association between serum and house dust (*r* = 0.34). Our multivariate models suggest that handwipes may at least partly serve as an intermediate variable between dust and serum levels. Watkins et al. (2011) observed a similar general pattern in their recent analysis of adults working in offices, although the associations presented here are much stronger, likely because toddlers have more hand-to-mouth activity than adults and spend more time in the home environment than office workers spend in their offices. Handwipes may also reflect exposure in more than one microenvironment and may be modified by handwashing (Watkins et al. 2011, 2012).

Our study suggests that handwipes may be a better matrix for examining exposure to contaminants found in dust than collecting dust itself, at least for the pentaBDE congeners evaluated in this study. Handwipes are potentially a more proximal measure of exposure than dust and may reflect exposure in more than one microenvironment. Handwipe samples are easier to collect and are more cost and time efficient than collecting dust. In addition, the best methods for collecting dust as a measure of exposure is not known; use of a less than optimal method should tend to diminish associations (Allen et al. 2008a). It may be easier to standardize collection of handwipes than to standardize collection of house dust given differences in vacuum cleaners used to collect dust, areas vacuumed, and differences in how dust is sieved and stored. Certainly standardized methods for collection and analysis of handwipes are needed, as is information on temporal variability in PBDE residues measured on the hands, perhaps through follow-up studies.

Our estimates suggest that pentaBDE body burdens increased with age in our cohort, by approximately 61%/year for ΣBDE_3_ and 73%/year for BDE-153. This might reflect both accumulation over time and/or increased hand-to-mouth activities with age. However, studies on children’s hand-to-mouth behavior are limited. Some data suggest that there are no differences in hand-to-mouth behavior over the age range of 1–4 years, whereas others have reported higher hand-to-mouth activity in children < 2 years of age compared with children 2–5 years of age (Black et al. 2005; Tulve et al. 2002).

ΣBDE_3_ concentrations in the serum of toddlers were inversely associated with parental education, after controlling for handwipe levels, child age, and sex (However, because race and SES are associated in our cohort, they are difficult to disentangle). Furthermore, we found no significant differences in PBDE dust concentrations by race or parental education (data not shown), suggesting that the exposure difference is not driven solely by higher levels of PBDEs in dust from homes with lower SES. Few predictors have been found for pentaBDE concentrations in dust. A previous study (Allen et al. 2008b) reported no correlation with counts of foam-containing furniture in Boston homes, but found a strong correlation when bromine concentrations (measured by X-ray fluorescence) were taken into account. The apparent SES-related differences in body burden of ΣBDE_3_ may be attributable to a number of factors that we did not take into account, including, for example, diet and differences in exposure patterns within or outside the home. More research is needed to understand the role of SES and race in determining exposure to ΣBDE_3_.

In marked contrast, BDE-153 in the serum of toddlers was positively associated with SES. However, this may at least be partly mediated by duration of breast-feeding, which is in turn associated with mother’s education in our population. The fact that BDE-153, but not ΣBDE_3_, was significantly associated with months of breast-feeding, combined with the lower correlations observed between BDE-153 in serum, handwipes, and dust levels, suggests that the sources and routes of exposure may differ between BDE 153 and BDEs 47, 99, and 100. Although BDEs 47, 99, 100 are commonly found in breast milk (Schecter et al. 2003; Wu et al. 2007), we did not find an association between serum ΣBDE_3_ and months of breast-feeding, suggesting that indoor exposure is more important for these congeners in toddlers. The difference may be attributable to a relative enrichment of BDE-153 to the other congeners in breast milk versus dust (we did not sample breast milk in our study). A comparison of our PBDE dust data to the data collected by Schecter et al. (2010) on PBDEs in breast milk does suggest that BDE-153 is more enriched in breast milk compared with BDE-47 when normalized to pentaBDE. Alternatively, this may also be attributable to a longer half-life for BDE-153 compared to the other BDE congeners. The human half-life of pentaBDE congeners has been only crudely estimated, not directly measured (Geyer et al. 2004). Similar to PCB-153, BDE-153 has a halogenation substitution pattern that results in low metabolic potential (Lupton et al. 2009). Therefore, serum levels of BDE-153 may be more reflective of cumulative exposures from *in utero,* infancy (e.g., breast milk), and dust exposures, whereas ΣBDE_3_ serum levels are likely attributable to more recent dust and dietary exposures.

Major strengths of our study include a relatively large sample size for an exposure study of PBDEs as well as concomitant measurement of blood, handwipes, and house dust. Potential weaknesses of our analysis include ignoring sources of dietary exposure and measurement of handwipes and dust at a single point in time. It is unknown how variable PBDE levels on handwipes are in a single person, although they do depend on frequency of handwashing (Watkins et al. 2011). In a previous study of Boston homes, we found that PBDEs in dust sampled 6–8 months apart were highly correlated (Allen et al. 2008a). Although the associations between PBDEs in serum, handwipes, and dust are most consistent with a hand-to-mouth exposure pathway following contact of hands with dust or PBDE-containing surface films, we cannot completely rule out dermal exposure, absorption of pentaBDEs from vapor to skin (Weschler and Nazaroff 2008), or reverse causation (in which PBDEs are excreted from skin), although some of these pathways appear to be unlikely (Watkins et al. 2011).

In conclusion, the results of our study suggest that toddlers have a significant amount of exposure to PBDEs from hand-to-mouth transfer. Children may also be exposed to other contaminants often found in house dust (e.g., polycyclic aromatic hydrocarbons, pesticides, perfluorinated compounds, other flame retardants) via hand-to-mouth activities. We also found striking differences in the relationships between SES and serum concentrations of different congeners. Although serum BDE-153 was positively associated with mother’s education, this association may be mediated by duration of breast-feeding. In contrast, BDEs 47, 99, and 100 were negatively associated with SES. Further research is needed to determine why SES factors are influencing PBDE exposure in children, which may help in mediating the potential health risks from exposure.

## Supplemental Material

(33 KB) PDF
