# Supplementary material for: Serum PBDEs in a North Carolina Toddler Cohort: Associations with Handwipes, House Dust, and Socioeconomic Variables
Source: Environ Health Perspect. 2012 May 23;120(7):1049–54. doi: 10.1289/ehp.1104802 (PMC3404669; doi:10.1289/ehp.1104802)
Supplement: (33 KB) PDF [file ehp.1104802.s001.pdf]

Supplemental Information

Serum PBDEs in a North Carolina Toddler Cohort: Associations with Hand Wipes, House Dust and Socioeconomic Variables

Heather M. Stapleton*[1], Sarah Eagle[1], Andreas Sjödin[2], and Thomas F. Webster[3]

1. Nicholas School of the Environment, Duke University, Durham, NC, USA

2. Centers for Disease Control and Prevention (CDC), National Center for Environmental Health (NCEH), Division for Laboratory Sciences (DLS), Atlanta, GA, USA

3. Dept. Environmental Health, Boston University School of Public Health, Boston, MA, USA

Address correspondence to:
Heather Stapleton, PhD
Nicholas School of the Environment
Duke University
LSRC, Box 90328
Durham, NC 27708
Phone: 919-613-8717
Fax: 919-684-8741
Email: heather.stapleton@duke.edu

Table S1. Pearson correlation coefficients for individual BDE congeners among serum, handwipes and dust.

		Log Serum					Log Handwipes					Log House Dust				
		BDE 47	BDE 99	BDE 100	BDE 153	ΣBDE_3	BDE 47	BDE 99	BDE 100	BDE 153	ΣBDE_3	BDE 47	BDE 99	BDE 100	BDE 153	ΣBDE_3
Log Serum	BDE47	1.000														
	BDE99	0.938$^{\#}$	1.000													
	BDE100	0.952$^{\#}$	0.902$^{\#}$	1.000												
	BDE153	0.606$^{\#}$	0.509$^{\#}$	0.692$^{\#}$	1.000											
	ΣBDE_3	0.997$^{\#}$	0.960$^{\#}$	0.962$^{\#}$	0.609$^{\#}$	1.000										
Log Hand wipes	BDE47	0.579$^{\#}$	0.587$^{\#}$	0.544$^{\#}$	0.401$^{\#}$	0.584$^{\#}$	1.000									
	BDE99	0.541$^{\#}$	0.596$^{\#}$	0.505$^{\#}$	0.305**	0.555$^{\#}$	0.943$^{\#}$	1.000								
	BDE100	0.567$^{\#}$	0.616$^{\#}$	0.528$^{\#}$	0.369**	0.580$^{\#}$	0.949$^{\#}$	0.962$^{\#}$	1.000							
	BDE153	0.482**	0.587$^{\#}$	0.480**	0.306**	0.510$^{\#}$	0.818$^{\#}$	0.857$^{\#}$	0.859$^{\#}$	1.000						
	ΣBDE_3	0.579$^{\#}$	0.608$^{\#}$	0.542$^{\#}$	0.370**	0.588$^{\#}$	0.987$^{\#}$	0.982$^{\#}$	0.971$^{\#}$	0.856$^{\#}$	1.000					
Log House Dust	BDE47	0.362**	0.301**	0.374**	0.256*	0.354**	0.388**	0.360**	0.333**	0.382**	0.374**	1.000				
	BDE99	0.314**	0.280*	0.323**	0.197	0.310**	0.338**	0.319**	0.285*	0.342**	0.330**	0.899$^{\#}$	1.000			
	BDE100	0.347**	0.300*	0.357**	0.258*	0.341**	0.348**	0.335**	0.300**	0.358**	0.340**	0.960$^{\#}$	0.909$^{\#}$	1.000		
	BDE153	0.302**	0.285*	0.312**	0.195	0.300**	0.301**	0.311**	0.267*	0.338**	0.301**	0.912$^{\#}$	0.908$^{\#}$	0.945$^{\#}$	1.000	
	ΣBDE_3	0.342**	0.291*	0.352**	0.227*	0.335**	0.372**	0.347**	0.310**	0.375**	0.359**	0.981$^{\#}$	0.961$^{\#}$	0.966$^{\#}$	0.942$^{\#}$	1.000

$^{*}p<0.05$; $^{**}p<0.01$, $^{\#}p<0.001$
